# Expression levels of interleukin-17 and interferon-γ in peripheral blood and relationship with thyroid function in patients with Hashimoto’s thyroiditis

**DOI:** 10.3389/fmolb.2025.1645736

**Published:** 2025-10-02

**Authors:** Zhiyong Huang, Jiaming Zhang, Si Chen, Mingfeng Mao, Boyong Wang

**Affiliations:** ^1^ Department of Clinical laboratory, The Central Hospital of Wuhan, Tongji Medical College, Huazhong University of Science and Technology, Wuhan, Hubei, China; ^2^ Department of Thyroid and Breast Surgery, The Central Hospital of Wuhan, Tongji Medical College, Huazhong University of Science and Technology, Wuhan, Hubei, China; ^3^ Department of the Third Operating Room, Zhongnan Hospital of Wuhan, Wuhan, Hubei, China; ^4^ Department of Medical Ultrasonics, The Central Hospital of Wuhan, Tongji Medical College, Huazhong University of Science and Technology, Wuhan, Hubei, China; ^5^ Department of the Thyroid and Breast Surgery, Zhongnan Hospital of Wuhan, Wuhan, Hubei, China

**Keywords:** Hashimoto’s thyroiditis, interleukin-17, interferon-gamma, thyroid function, thyroidautoantibodies

## Abstract

**Objective:**

This study aims to unveil the interleukin-17 (IL-17) and interferon-gamma (IFN-γ) levels in the peripheral blood of patients with Hashimoto’s thyroiditis (HT) and their association with thyroid function.

**Methods:**

We selected 68 HT patients admitted to our hospital and 36 healthy individuals undergoing physical examinations as controls. Clinical data were collected, and serum IL-17 and IFN-γ levels were measured. The Pearson method was used to analyze the correlations between IL-17 and IFN-γ levels and thyroid function parameters. A logistic regression analysis model was employed to evaluate the influencing factors of HT. ROC curves were utilized to assess the diagnostic value of IL-17 and IFN-γ for the occurrence of HT.

**Results:**

Serum IL-17 and IFN-γ levels were higher in both HT patients with hypothyroidism and those without hypothyroidism compared to healthy controls (*P* < 0.05). IL-17 levels demonstrated a positive correlation with TGAb, TPOAb, and TSH among HT patients, while showing a negative correlation with FT4 (*P* < 0.05). Similarly, IFN-γ levels were positively related to TGAb and TPOAb (*P* < 0.05). IL-17 and IFN-γ levels were identified as influencing factors for the occurrence of HT (OR: 1.012, 1.028; *P* < 0.05). The optimal cutoff values for distinguishing HT from healthy controls were >629.77 pg/mL for IL-17, >286.04 ng/L for IFN-γ, and (>683.02 pg/mL, >252.73 ng/L) for the combination of both. The areas under the curves were 0.854 (0.771–0.936), 0.795 (0.697–0.894), and 0.903 (0.846–0.960), respectively.

**Conclusion:**

Serum IL-17 and IFN-γ levels are highly expressed in HT patients, and both are closely related to thyroid function and autoantibody levels. They possessed a certain value for the early diagnosis of HT.

## Introduction

Hashimoto’s thyroiditis (HT) is a chronic autoimmune disorder characterized by lymphocytic infiltration of the thyroid gland, elevated thyroglobulin antibody (TGAb) and thyroid peroxidase antibody (TPOAb), along with progressive thyroid dysfunction (Ihnatowicz et al., 2020). It represents the most common reason for hypothyroidism in iodine-sufficient regions, affecting approximately 10%–12% of the global population, with a striking female predominance (Mikulska et al., 2022). The disease is marked by the presence of thyroid-specific autoimmunity, where immune-mediated destruction of thyrocytes leads to impaired hormone synthesis and eventual gland failure (Lebiedzinski and Lisowska, 2023). The pathogenesis of HT involves a complex interplay of genetic predisposition, environmental triggers, alongside dysregulated immune responses (Song et al., 2025). Central to this process is the imbalance between pro-inflammatory T helper cell subsets, particularly T helper (Th) 1 and Th17, and impaired regulatory T cell function (Fang et al., 2022). Th1 cells secrete interferon-gamma (IFN-γ), a key cytokine that drives cytotoxic immune responses and enhances thyroid follicular cell damage (Clark et al., 2022). IFN-γ signaling through the JAK-STAT pathway induces the expression of pro-inflammatory mediators, driving thyroid inflammation (Coelho et al., 2023).

Concurrently, Th17 cells, which produce interleukin-17 (IL-17), exacerbate tissue inflammation by recruiting neutrophils and amplifying immune-mediated thyroid injury (Wang et al., 2022). IL-17 acts synergistically with other cytokines, such as TNF-α, to disrupt thyroid follicular integrity and promote autoantibody production (Zhao et al., 2021). Elevated serum IL-17 levels have been strongly associated with autoimmune diseases, including HT, where they correlate with disease severity and autoantibody titers (Huangfu et al., 2023). Additionally, a diminished Treg response further contributes to immune dysregulation, allowing unchecked Th1 and Th17 activity (Fang et al., 2022). Previous studies have demonstrated significant elevations in IFN-γ and IL-17 in HT patients, suggesting their critical roles in disease progression (Tywanek et al., 2024). IFN-γ not only promotes macrophage polarization toward a pro-inflammatory M1 phenotype but also enhances antigen presentation, sustaining autoimmune responses (Fu et al., 2023). Similarly, IL-17 disrupts immune tolerance by stimulating the release of secondary inflammatory cytokines and chemokines, exacerbating thyroid tissue damage (Liu et al., 2022). However, the exact relationship between these cytokines and thyroid dysfunction—particularly their influence on thyroid hormone levels, such as triiodothyronine (FT3), free thyroxine (FT4), along with thyroid-stimulating hormone (TSH)—remains incompletely understood (Kryczyk-Koziol et al., 2022). This paper aims to evaluate the IL-17 and IFN-γ levels in the peripheral blood of HT patients and analyze their correlations with thyroid function parameters and autoantibody levels. By elucidating these relationships, we seek to clarify the immunopathogenic mechanisms of HT and identify potential biomarkers for disease monitoring and therapeutic intervention.

## Materials and methods

### Ethical approval

All experimental procedures were ratified by the Medical Ethics Committee of The Central Hospital of Wuhan, Tongji Medical College, Huazhong University of Science and Technology and were conducted in accordance with the Declaration of Helsinki.

### Subjects

We selected 68 patients with HT admitted to The Central Hospital of Wuhan, Tongji Medical College, Huazhong University of Science and Technology from June 2023 to June 2024 as the study subjects, who were divided into the HT non-hypothyroidism group (23 cases) and the HT hypothyroidism group (45 cases) based on their thyroid function. Among the 68 HT patients, there were 20 males and 48 females, aged between 25 and 61 years, with an average age of 42.76 ± 9.17 years. Their body mass index (BMI) was 23.49 ± 3.05 kg/m^2^. The inclusion criteria were: meeting the diagnostic criteria for HT (Klubo-Gwiezdzinska and Wartofsky, 2022); no prior treatment with thyroid hormones or other medications affecting thyroid function. The exclusion criteria were: concurrent severe heart, liver, or kidney diseases; recent pathological conditions that could affect the concentrations of inflammatory markers (such as various acute or chronic infections, etc.); concurrent malignant tumors; concurrent mental dysfunction; pregnant or lactating women. We selected 36 healthy volunteers who underwent physical examinations at our hospital during the same period as the control group. The control group had no history of thyroid diseases, hypertension, diabetes, coronary heart disease, mental disorders, malignant tumors, autoimmune diseases, or family history. In the control group, there were 9 males and 27 females, aged 24–63 years (mean: 42.83 ± 10.11) years, with a BMI of 22.45 ± 2.60 kg/m^2^.

### Specimen collection and processing

Peripheral venous blood was obtained from all participants in the morning after fasting. The samples were centrifuged for 10 min, followed by serum separation and preservation at −20 °C for further analysis.

### IL-17 and IFN-γ levels measurement

Serum IL-17 and IFN-γ levels were tested using ELISA kits (Rapidbio, United States), and the DNM-9602 microplate reader from Perlong (Beijing, China) was employed for the measurements.

### Thyroid autoantibody levels detection

Serum levels of TGAb and TPOAb were estimated using electrochemiluminescence immunoassay. The measurements were executed using the COBAS E411 fully automated chemiluminescence analyzer (Roche, Germany).

### Thyroid function parameter measurement

Serum levels of FT3, FT4, total triiodothyronine (TT3), total thyroxine (TT4), and TSH were measured using electrochemiluminescence immunoassay with Roche COBAS E411 fully automated chemiluminescence analyzer.

### Statistical analysis

A *post hoc* analysis was conducted using Gpower software (version 3.1) based on the differences in serum IL-17 and IFN-γ levels between the control group and the HT groups, with an alpha error level of 0.05, resulting in a power of 1-beta exceeding 0.95 for both. Data analysis was processed SPSS 21.0 software (IBM Corp, Armonk, N.Y, United States) coupled with GraphPad Prism 6.01 software (GraphPad Inc., La Jolla, CA, United States). For continuous variables, data were expressed as mean ± standard deviation (SD). Differences among multiple groups were evaluated by one-way ANOVA, with pairwise comparisons conducted via the LSD-t test. Categorical variables were described as percentages, and intergroup differences were analyzed using the chi-square (χ^2^) test. The correlation between variables was determined by the Pearson test. The Receiver Operating Characteristic (ROC) curve was employed to determine the diagnostic value of each indicator for the occurrence of HT, and binary logistic regression analysis was performed to identify relevant factors influencing the occurrence of HT. A *P*-value less than 0.05 was considered statistically significant in all tests.

## Results

### Serum IL-17 and IFN-γ levels

The serum IL-17 and IFN-γ levels in both the HT hypothyroidism group and the HT non-hypothyroidism group were higher than those in the control group (*P* < 0.05). The serum IL-17 and IFN-γ levels in the HT hypothyroidism group were higher than those in the HT non-hypothyroidism group (*P* < 0.05). Serum IL-17 and IFN-γ levels are highly expressed in HT patients, suggesting their potential involvement in the pathogenesis of HT (Table 1).

**TABLE 1:** Comparison of serum IL-17 and IFN-γ levels among the three groups (mean ± SD).

Index	Control group (n = 36)	HT non-hypothyroidism group (n = 23)	HT hypothyroidism group (n = 45)	*F*	*P*
IL-17 (pg/mL)	534.21 ± 129.28	658.30 ± 98.12^*^	725.70 ± 77.32^*#^	23.62	<0.001
IFN-γ(ng/L)	247.55 ± 57.24	285.77 ± 41.17^*^	314.41 ± 30.52^*#^	14.93	<0.001

Compared with the control group, *P < 0.05; compared with the HT non-hypothyroidism group, ^#^ *P* < 0.05. IL-17, interleukin-17; IFN-γ, interferon-gamma; HT, Hashimoto’s thyroiditis.

### Serum thyroid autoantibody levels

The serum TGAb and TPOAb levels in both the HT with hypothyroidism group and the HT without hypothyroidism group were higher relative to the control group. Additionally, the HT with hypothyroidism group presented higher serum TGAb and TPOAb levels relative to the HT without hypothyroidism group (*P* < 0.05). The autoantibody levels were elevated in both hypothyroid and euthyroid HT patients (Table 2).

**TABLE 2:** Comparison of serum thyroid levels among three groups (mean ± SD).

Index	Control group (n = 36)	HT non-hypothyroidism group (n = 23)	HT hypothyroidism group (n = 45)	*F*	*P*
TGAb (IU/mL)	0.08 ± 0.28	160.91 ± 28.46^*^	184.73 ± 32.98^*#^	571.059	<0.001
TPOAb (IU/mL)	1.22 ± 1.02	277.52 ± 71.98^*^	343.76 ± 81.54^*#^	307.962	<0.001

Compared with the control group, *P < 0.05; compared with the HT non-hypothyroidism group, ^#^ *P* < 0.05. TGAb, thyroglobulin antibody; TPOAb, thyroid peroxidase antibody; HT, Hashimoto’s thyroiditis.

### Serum thyroid function parameters

Serum levels of FT3, FT4, TT3, and TT4 were lower in HT patients with hypothyroidism in contrast to both the HT non-hypothyroidism group and healthy controls, whereas TSH levels were elevated (*P* < 0.05). No significant differences in these thyroid function parameters were witnessed between the HT non-hypothyroidism group and the control group (*P* > 0.05). Thyroid hormone depletion predominantly occurs in HT patients with hypothyroidism, while those without hypothyroidism maintain thyroid hormone levels comparable to healthy individuals (Table 3).

**TABLE 3:** Comparison of serum thyroid function parameters among the three groups (mean ± SD).

Index	Control group (n = 36)	HT non-hypothyroidism group (n = 23)	HT hypothyroidism group (n = 45)	*F*	*P*
FT3 (pg/mL)	2.54 ± 0.55	2.59 ± 0.63	1.29 ± 0.43^*#^	76.059	<0.001
FT4 (ng/dL)	1.38 ± 0.38	1.41 ± 0.52	0.60 ± 0.12^*#^	69.118	<0.001
TT3 (ng/mL)	1.51 ± 0.41	1.54 ± 0.44	0.56 ± 0.10^*#^	113.415	<0.001
TT4 (μg/dL)	8.98 ± 3.24	9.05 ± 3.32	2.76 ± 0.97^*#^	77.103	<0.001
TSH (μIU/mL)	2.55 ± 0.68	2.59 ± 0.79	28.10 ± 8.50^*#^	262.088	<0.001

*P < 0.05 compared with the control group; ^#^ *P* < 0.05 compared with the HT non-hypothyroidism group. FT3, triiodothyronine; FT4, free thyroxine; TT3, total triiodothyronine; TT4, total thyroxine; TSH, thyroid-stimulating hormone; HT, Hashimoto’s thyroiditis.

### Correlation between IL-17 and IFN-γ expression levels in HT patients

Pearson correlation analysis reflected a positive link between serum IL-17 and IFN-γ levels (r = 0.451, *P* < 0.001). These results indicate a certain correlation between IL-17 and IFN-γ expression levels in HT patients.

### Correlation of IL-17 and IFN-γ expression levels with thyroid function in HT patients

Pearson correlation analysis revealed that serum IL-17 levels had a positive connection with TGAb, TPOAb, and TSH levels, and a negative connection with FT4 levels (*P* < 0.05), while no correlation was observed with FT3, TT3, or TT4 (*P* > 0.05). Serum IFN-γ levels were positively related to TGAb and TPOAb levels (*P* < 0.05), but showed no correlation with FT3, FT4, TT3, TT4, or TSH (*P* > 0.05). Levels of IL-17 and IFN-γ in HT patients are closely related to thyroid function and autoantibody levels, potentially participating in the immune dysregulation process of the thyroid (Table 4).

**TABLE 4:** Correlation between IL-17, IFN-γ expression levels and thyroid function in HT patients.

Indicator	IL-17		IFN-γ	
	*r*	*P*	*r*	*P*
TGAb	0.477	<0.001	0.257	0.034
TPOAb	0.510	<0.001	0.282	0.020
FT3	−0.140	0.256	−0.088	0.475
FT4	−0.352	0.003	−0.174	0.156
TT3	−0.002	0.987	0.081	0.514
TT4	−0.028	0.818	0.080	0.517
TSH	0.318	0.008	0.230	0.060

IL-17, interleukin-17; IFN-γ, interferon-gamma; HT, Hashimoto’s thyroiditis; TGAb, thyroglobulin antibody; TPOAb, thyroid peroxidase antibody; FT3, triiodothyronine; FT4, free thyroxine; TT3, total triiodothyronine; TT4, total thyroxine; TSH, thyroid-stimulating hormone.

### Multivariate analysis

Whether HT occurred (1 = HT, 0 = healthy control) was used as the dependent variable, with gender, age, BMI, serum IL-17, and IFN-γ levels as independent variables included in a binary logistic regression model for analysis. The results (Table 5) showed that high levels of IL-17 (OR: 1.012, 95% CI: 1.006–1.019, *P* < 0.001) and IFN-γ (OR: 1.028, 95% CI: 1.010–1.046, *P* = 0.002) were independent risk factors for HT.

**TABLE 5:** Logistic regression analysis of influencing factors for the onset of HT.

Variable	B	Standard error	Wald	*P*	OR	95%CI
Gender	0.935	0.710	1.736	0.188	2.548	0.634–10.245
Age	−0.047	0.043	1.176	0.278	0.954	0.876–1.039
BMI	0.065	0.116	0.308	0.579	1.067	0.849–1.34
IL-17	0.012	0.003	13.826	<0.001	1.012	1.006–1.019
IFN-γ	0.027	0.009	9.668	0.002	1.028	1.01–1.046

HT, Hashimoto’s thyroiditis; BMI, body mass index; IL-17, interleukin-17; IFN-γ, interferon-gamma; OR, odds ratio; CI, confidence interval.

### ROC analysis of the diagnostic value of IL-17 and IFN-γ levels for HT

**FIGURE 1:**
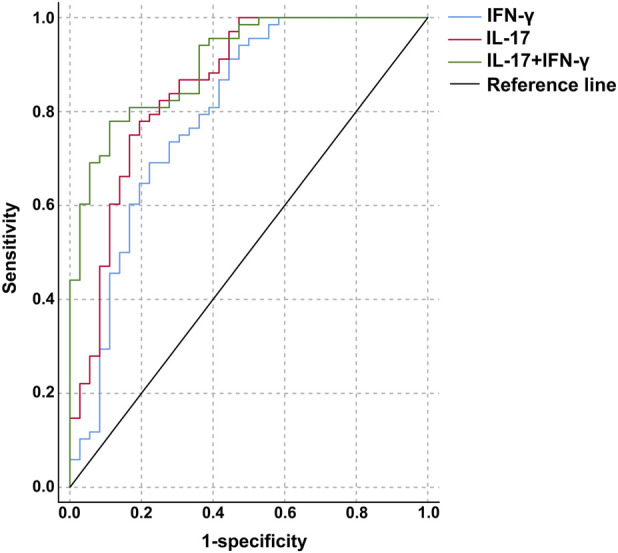
ROC curve for the diagnosis of HT based on the levels of IL-17 and IFN-γ.

The ROC curve was used to analyze the diagnostic value of IL-17 and IFN-γ levels for HT. The optimal cutoff value for IL-17 was 629.77 pg/mL, with an area under the curve of 0.854 (0.771–0.936), and the sensitivity and specificity for distinguishing the HT groups from the control group were 77.9% and 80.6%, respectively. The optimal cutoff value for IFN-γ was 286.04 ng/L, with an area under the curve of 0.795 (0.697–0.894), and the sensitivity and specificity for distinguishing the HT groups from the control group were 69.1% and 77.8%. The combined application of IL-17 and IFN-γ with optimal cutoff values of >683.02 pg/mL and >252.73 ng/L, yielded an area under the curve of 0.903 (0.846–0.960), with sensitivity and specificity of 77.9% and 88.9% (Table 6; Figure 1).

**TABLE 6:** Diagnostic value of IL-17 and IFN-γ for HT.

Variable	AUC (95% CI)	*P*	Cut-off value	Youden’s index	Sensitivity (%) |	Specificity (%)
IL-17	0.854 (0.771–0.936)	<0.001	>629.77 pg/mL	0.585	77.90%	80.60%
IFN-γ	0.795 (0.697–0.894)	<0.001	>286.04 ng/L	0.469	69.10%	77.80%
IL-17 + IFN-γ	0.903 (0.846–0.960)	<0.001	>683.02 pg/mL,>252.73 ng/L	0.668	77.90%	88.90%

HT, Hashimoto’s thyroiditis; IL-17, interleukin-17; IFN-γ, interferon-gamma; AUC, area under the curve; CI, confidence interval.

## Discussion

HT is a chronic autoimmune disorder featured with lymphocytic infiltration, destruction of thyroid tissue, and the presence of thyroid autoantibodies, such as TPOAb and TGAb (Knezevic et al., 2020). The pathogenesis of HT involves a complex interplay of pro-inflammatory cytokines, including IL-17 and IFN-γ, which are known to play critical roles in autoimmune responses (Zhang et al., 2021). In this study, we observed elevated IL-17 and IFN-γ levels in HT patients, which were closely related to thyroid function and autoantibody levels.

IL-17 and IFN-γ contribute to the inflammatory milieu in autoimmune thyroid diseases by promoting thyroid tissue damage and altering thyroid hormone levels (Liu et al., 2022). Compared to the control group, serum levels of IL-17 and IFN-γ were higher in HT patients in this study, with higher levels in the HT hypothyroidism group than in the non-hypothyroidism group. This suggests that IL-17 and IFN-γ may be involved in HT pathogenesis and play different roles at different disease stages (with or without hypothyroidism). As previous studies have reported increased IL-17 expression in HT peripheral blood (Peng et al., 2021) and a positive correlation between IFN-γ and thyroid autoantibodies (Kryczyk-Koziol et al., 2022), Additionally, high levels of IL-17 and IFN-γ were independent risk factors for HT, further confirming their importance in HT pathogenesis. They may promote thyroid tissue damage and inflammation by regulating the immune response, leading to HT onset and progression.

Meanwhile, serum TGAb and TPOAb levels were higher in both the hypothyroidism and non-hypothyroidism groups compared to the control group, with higher levels in the hypothyroidism group, showing a certain similarity to the trends of IL-17 and IFN-γ levels. Positive correlations were found between serum IL-17 levels and TGAb/TPOAb levels, as well as between serum IFN-γ levels and TGAb/TPOAb levels, indicating that IL-17 and IFN-γ may promote the production of thyroid autoantibodies by regulating the immune system. Thyroid autoantibodies (TGAb and TPOAb), important markers of HT, attack thyroid tissue, causing damage and destruction of thyroid cells, and thus affecting thyroid function (Luo et al., 2025). IL-17 and IFN-γ may increase the secretion of thyroid autoantibodies by activating B lymphocytes and promoting their differentiation into plasma cells. The association of higher IFN-γ levels with elevated TGAb/TPOAb resonates with studies implicating IFN-γ in thyrocyte apoptosis and antigen presentation (Lechner et al., 2023), which may drive antibody production.

Compared to the HT non-hypothyroidism group and healthy control group, HT hypothyroidism patients had lower serum FT3, FT4, TT3, and TT4 levels and higher TSH levels, consistent with a hypothyroid state. The positive association between serum IL-17 levels and TSH and the negative association with FT4 suggest that IL-17 may regulate TSH levels by affecting thyroid hormone synthesis and secretion or promote thyroid tissue damage and inhibit hormone synthesis. In contrast, serum IFN-γ levels showed no correlation with thyroid function indicators, indicating that its role in regulating thyroid function differs from that of IL-17 or involves a more complex mechanism that does not directly participate in hormone synthesis and secretion regulation. Biologically, IL-17 may more directly affect thyroid cell function and autoantibody production through multiple pathways, while IFN-γ, although involved in immune regulation, may mainly regulate the differentiation and functional balance of immune cells, with a relatively weaker direct impact on thyroid cells and autoantibodies. Here, we extend this understanding by demonstrating that IFN-γ correlates more strongly with autoantibodies than with thyroid dysfunction itself. Conversely, IL-17’s dual correlation with autoantibodies and TSH mirrors findings in other autoimmune diseases where IL-17 promotes tissue inflammation (Chen et al., 2024).

Serum IL-17 and IFN-γ levels show a positive correlation, indicating a possible synergistic role in HT pathogenesis. They may jointly regulate immunity, activate immune cell subsets, amplify responses, and worsen thyroid tissue damage and inflammation. High levels of both are independent HT risk factors, increasing disease risk independently of other confounding factors. Further research shows that exceeding cut-off values for either cytokine suggests a higher HT likelihood, aiding preliminary screening and diagnosis. Combined cytokine detection is more effective than individual tests, improving diagnostic accuracy and sensitivity, reducing missed and misdiagnoses, and enabling early, precise HT diagnosis. Evidence shows that IL-17 secretion contributes to epithelial homeostasis, acute inflammatory responses, and B-cell stimulation after appropriate stimulation, acting as a bridge between innate and acquired immune responses (Li et al., 2022). Shuiping Li et al. found that serum IL-17 levels may represent a new biomarker for diagnosing malignant HT nodules (Li et al., 2022). An animal experiment reported elevated IFN-γ and IL-17A levels, as well as increased concentrations of IFN-γ and IL-17, in HT mice (Liu et al., 2024). Importantly, in thyroid-specific IFN-γ transgenic mice, IFN-γ plays a direct role in autoimmune thyroid destruction (Wu et al., 2022).

In conclusion, serum IL-17 and IFN-γ levels are highly expressed in HT patients and are closely related to thyroid function and autoantibody levels, holding certain value for the early diagnosis of HT. This suggests that serum IL-17 and IFN-γ levels can assist in early diagnosis, assess disease severity, and may provide references for treatment decisions and prognosis judgment, facilitating personalized medicine. This study had a limited sample size, which may affect the generalizability of our findings. Additionally, without comparing with additional autoimmune or inflammatory disease groups, it is difficult to determine whether elevated serum IL-17 and IFN-γ levels are unique to HT or common features of other autoimmune or inflammatory diseases. Furthermore, as a cross-sectional study, this research can only reflect the relationships between serum IL-17 and IFN-γ levels and thyroid function and autoantibody levels at a single time point, unable to determine causal relationships and time sequences. Given the aforementioned limitations, future research can expand the sample size and conduct multi-center prospective studies to monitor changes in cytokines and thyroid function over time, helping to clarify causal relationships and time sequences. Moreover, comparing with other autoimmune or inflammatory disease groups in future studies can determine the specificity of serum IL-17 and IFN-γ levels in HT. Based on in-depth research on the mechanisms of serum IL-17 and IFN-γ in HT pathogenesis, targeted therapy studies on these cytokines can be carried out to provide new treatment options and better treatment strategies for HT patients.

## Data Availability

The original contributions presented in the study are included in the article/supplementary material, further inquiries can be directed to the corresponding authors.
